# Visual quality observation of clear lens extraction by ultrasonic phacoemulsification and intraocular lens implantation in a child with microspherophakia: A case report

**DOI:** 10.1097/MD.0000000000021937

**Published:** 2020-08-21

**Authors:** Qian Liu, Xiaogang Wang, Suhua Zhang

**Affiliations:** Cataract Department, Shanxi Eye Hospital, Taiyuan, Shanxi Province, China.

**Keywords:** iTrace, microspherophakia, visual quality

## Abstract

**Rationale:**

Microspherophakia is characterized by a small, spherical crystalline lens with increased sagittal diameter. Because of the uncertainty about the outcome, as well as the complexity of the operation and development of complications, the management and timing of surgical intervention for microspherophakia are still debated. Lens extraction is effective for avoiding the risk of pupillary blockage, but the outcome after operation is controversial. The iTrace (Tracey, USA) report shows the influence of low-order aberrations (LOA) and high-order aberrations (HOA), which may be valuable in predicting postoperative outcome. Our report concerns a child with microspherophakia who underwent lens extraction via the analysis of visual quality by iTrace.

**Patient concerns:**

Our report is on the case of a 7-year-old girl whose parents observed she had to bring her papers and books extremely close to her face to read. On examination, the girl was bilaterally diagnosed microspherophakia with a small tremble lens. The objective refraction was −15.0 diopter of spherical power (DS)/−1.00 diopter of cylindrical power (DC) × 180 right eye (OD) and −12.5 DS/−1.50 DC × 20 left eye (OS). The HOA of OD and OS were high up to 0.926 and 0.659, respectively by iTrace. The visual quality remained terrible after correcting LOA (high myopia and astigmatism). According to iTrace report, the patient would get a good visual quality by extracting the clear lens with HOA from cornea after correcting LOA. The girl's parent opted for surgery on the left eye.

**Diagnosis:**

Due to the patient's symptoms, examination results, she was diagnosed with microspherophakia.

**Interventions:**

The patient underwent clear lens extraction by ultrasonic phacoemulsification and intraocular lens implantation.

**Outcomes:**

The first day after operation, total HOA was decreased to 0.077. Total LOA was 0.713. Corrected distance visual acuity (CDVA) is 20/20. One week after surgery, HOA was 0.110 and LOA was 0.328. CDVA was 20/25. CDVA was still 20/25 one month after surgery. The total HOA was 0.110 and the LOA was 0.334 by iTrace.

**Lessons:**

ITrace not only plays an important role in analyzing potential reasons of undesirable preoperative visual quality but also can predict postoperative outcomes. All these functions are helpful for determining surgical intervention of microspherophakia cases.

## 1 Introduction

Microspherophakia is a rare congenital anomaly of lens which appears asbilateral spherophakia and microphakia.^[1,2]^ The characteristic feature of microspherophakia is a small lens diameter.^[2–5]^ High lenticular myopia often occurs. Tremor of lens with changes in posture and the lens equator is visible on full mydriasis.^[1]^ Glaucoma is the main complication, affecting up to 51% of eyes with microspherophakia and leading, in some cases, to visual impairment. The pathogenesis of this condition is thought to be pupillary block by spherical lens,^[6]^ which is associated with defective development of the lens zonules.^[7]^ Lens dislocation or subluxation is a common symptom, leading to defective accommodation.^[1]^ Treatment of microspherophakia is difficult and controversial. In this article, we report a case of a subject who underwent clear lens extraction and intraocular lens implantation by the guide of iTrace, and the patient gain a good visual quality postoperatively.

This case report was approved by the ethics committee of the Shanxi Eye Hospital, Taiyuan, China, and the informed consent form was signed by patient.

## 2 Case presentation

This study was allowed by the Institutional Review Board of Shanxi Eye Hospital and complied with the tenets of the Declaration of Helsinki. Written informed consent was obtained from the patient's father for publication of this case report and any accompanying images.

**Figure 1:**
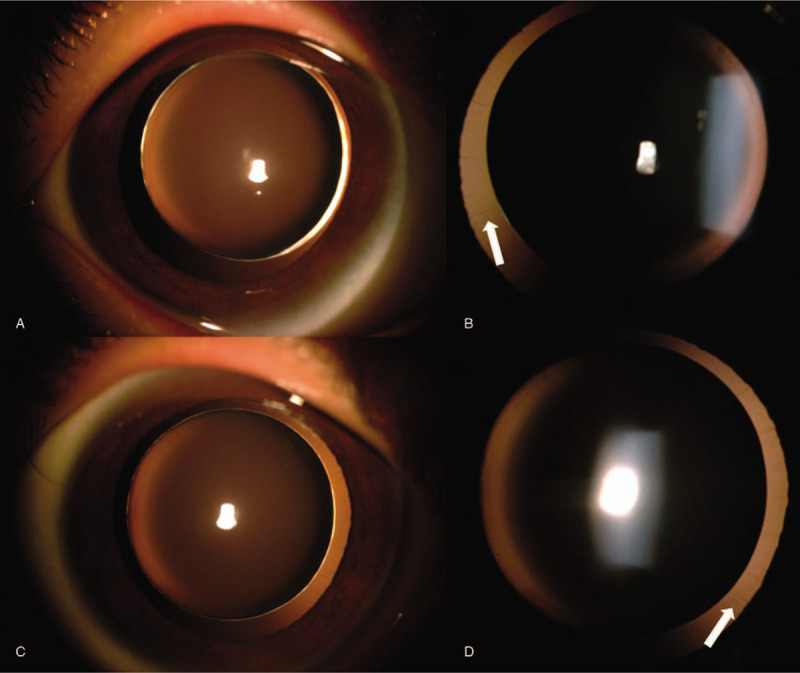
(A) and (B) show right eye and left eye of this patient, respectively. Boundary and form of lens are apparent. (C) and (D) show the zonules of 2 eyes, indicated by the white arrow.

**Figure 2:**
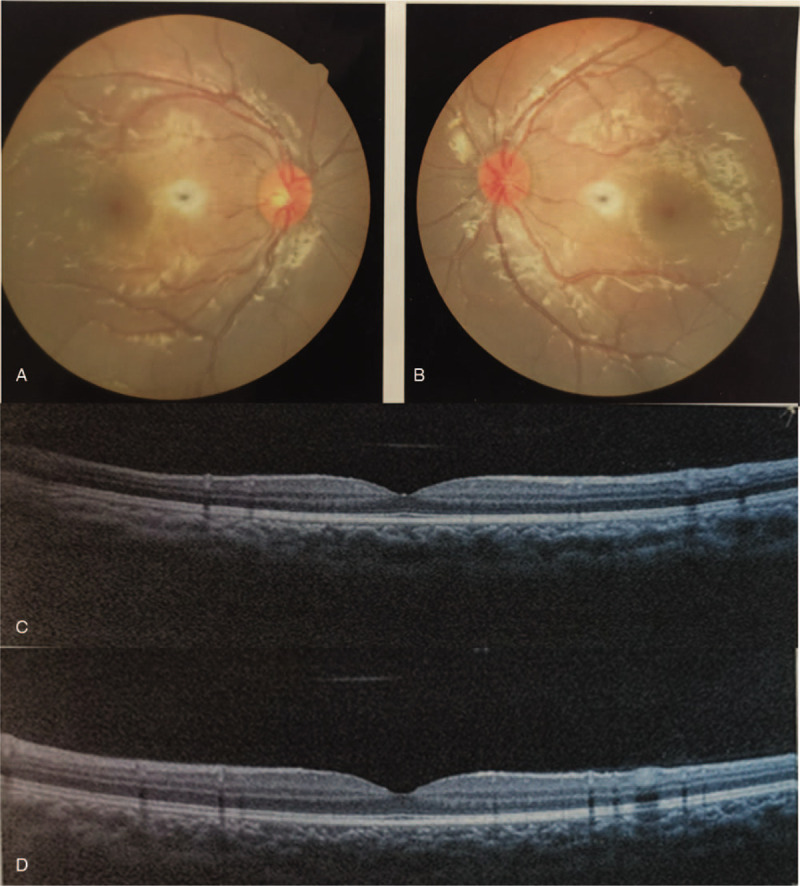
(A) and (B) are fundus photographs of right eye (OD) and left eye (OS). (C) and (D) are optical coherence tomography of OD and OS, respectively. No obvious abnormities appear in the photographs.

A 7-year-old girl came to our hospital with her parents, who stated that their daughter would have to look extremely close when writing and reading for 1 year. Her visual acuity was 20/400 with −15.0 DS/−1.00 DC × 180 in the right eye (OD) and 20/125 with −12.5 DS/−1.50 DC × 20 in the left eye (OS). Intraocular pressure (IOP) was 15 mmHg in OD and 18 mmHg in OS. Slit-lamp biomicroscopy showed a deep anterior chamber that protruded forward in the pupillary zone of the iris in both eyes. The iris and lens trembled with changes in posture. As for the lens edges and form, weak zonules were clearly visible on slit-lamp examination after pupillary dilation (Fig. 1). The equatorial diameters were 6.20 mm OD and 6.30 mm OS by color ultrasound. Lens thicknesses were 5.07 mm OD and 5.05 mm OS. Central corneal thickness was 548 μm OD and 547 μm OS. Axial length was 22.97 mm OD and 22.61 mm OS. The data above were acquired with Lenstar 900(Haag-Streit Diagnostics, Switzerland). The anterior chamber depth was 3.53 mm OD and 3.40 mm OS via MASTER (ZEISS. Germany). Fundus color camera and Heidelberg retinal tomography revealed no apparent abnormalities (Fig. 2). A thorough family history could not be obtained. No systemic anomalies were found on detailed pediatric examination. Mental status was within normal limits for both siblings.

**Figure 3:**
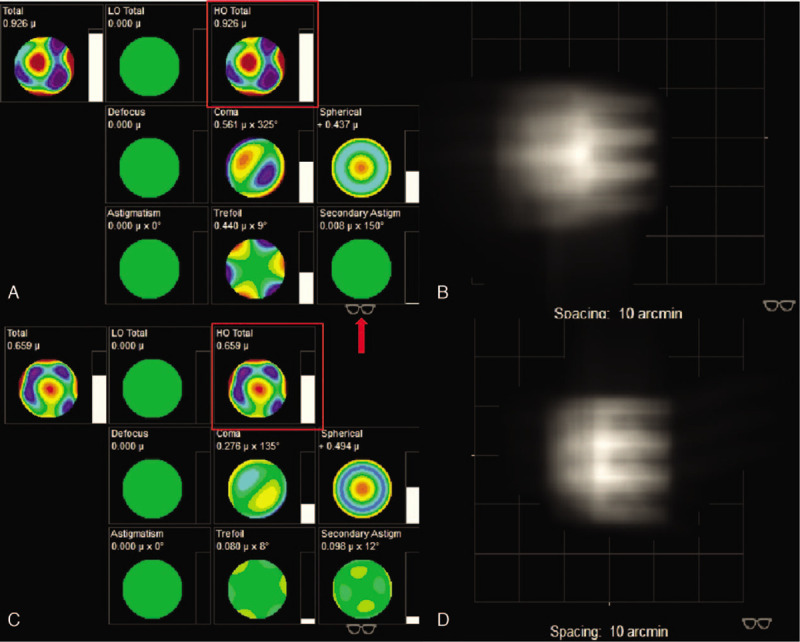
The iTrace report of the patient. (A and C) Show the low-order aberration and high-order aberration (HOA) of right eye (OD) and left eye (OS), respectively. (B and C) Show the visual quality of OD and OS, respectively. Total HOA of OD is 0.926, marked by the red square in A. Total HOA of OS is 0.659, marked by the red square in B. The identification marked by the red arrow indicates status correcting LOA (highmyopia and astigmatism).

**Figure 4:**
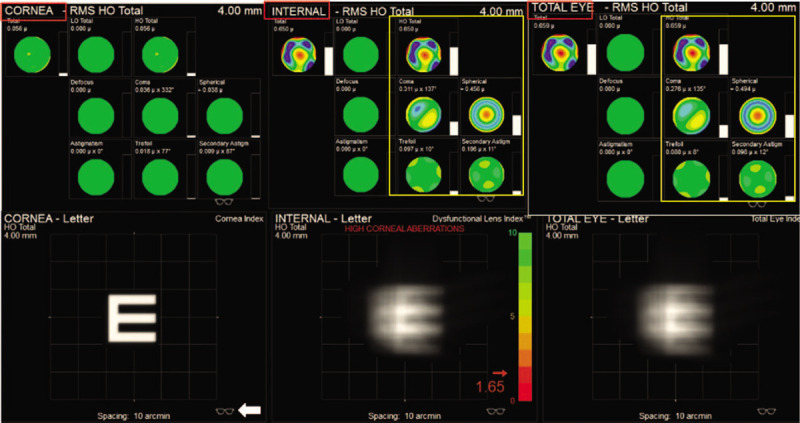
The preoperative iTrace report showed high-order aberrations (HOA) of cornea, internal respectively marked by red box and the influence on visual quality of total eye. HOA of total eye was mainly from internal eye marked by yellow box. The visual quality was good without the influencing factor of internal eye which showed as the optotype effected only by cornea factor after the high-order aberrations was corrected as the white arrow showed.

Although the girl's high myopia and astigmatism seriously affected her quality of life, high-order aberrations (HOA) were still high up to 0.926 OD and 0.659 OS after correcting high myopia and astigmatism. The optotype appeared still in poor visual quality after correcting low-order aberrations (LOA) (Fig. 3). In consideration of postoperative outcomes and surgical risks, the girl's parents chose the left eye for treatment. The mainly potential source of undesirable vision OS came from internal HOA after correcting LOA, which could not be solved by conservative treatment such as wearing glasses showed in Figure 4. The fundus examination showed no obvious abnormity (Figure 2). Therefore, good postoperative visual outcomes was expected to acquire by removing the microspherophakia , which demonstrated using the iTrace. The residual influence factor was mainly from cornea which significantly reduced the influence on visual quality with total HOA as 0.056 showed in Figure 4. In view of general anesthesia for the child, surgery assisted by femtosecond laser was abandoned. As a result, the treatment of ultrasonic phacoemulsification and intraocular lens (IOL) implantation for OS was decided by the parents. The chosen IOL was AR40e (A-constant 118.4) (Abbott Medical Optics Inc., Santa Ana, CA). IOL power was+27.00 D for a residual refraction of −0.48 D by SRK-T formula and +0.11D by Barrette Universal II formula.

The operation was carried out under a general anesthetic. Initially, to reduce surgically induced corneal astigmatism, a 3.0-mm scleral tunnel incision was made at the 11 o’clock position and an accessory 15-degree blade limbus incision was made at the 2 o’clock position. An approximately 4 mm capsulorhexis and phacoemulsification were performed. The operation was extremely difficult because of the extremely loose zonules. A hydrophobic acrylic 3-piece IOL of +25.00 D power Sensar AR40e was implanted in the sulcus. To ensure that the optic region was centered, the margin of IOL was incarcerated by anterior capsule opening. After clearing the viscoelastic agent, a scleral tunnel incision was seamed by 10–0 black monofilament nonabsorbable suture.

**Figure 5:**
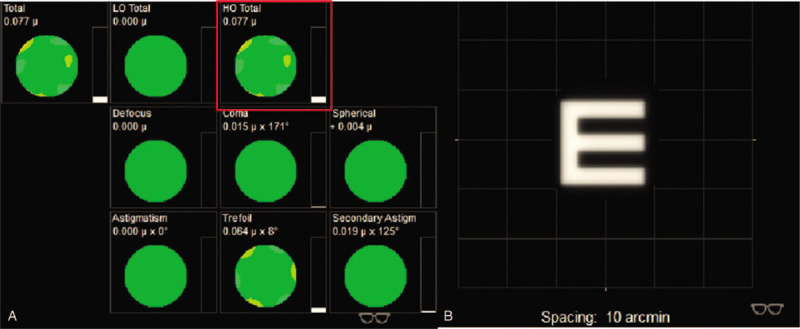
The iTrace report on the first day after operation, with status of correcting high-order aberrations. Total high-order aberrations is 0.077, marked by the red square. (B) Shows a clear visual, obtained by wearing glasses.

**Figure 6:**
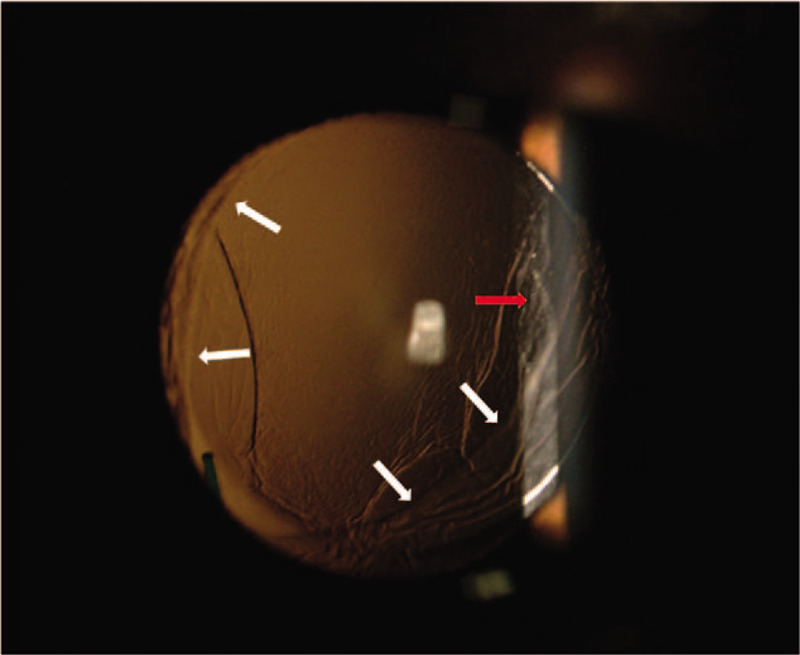
A colour photo of the anterior segment after mydriasis, 1 month post-operation. The intraocular lens (IOL) was incarcerated by anterior capsule opening, as the white arrows show. Capsule regrowth is marked by the red arrow. The IOL remained centred in the pupil region.

On the first day after operation, the girl had an uncorrected visual acuity of 20/40. The corrected distance visual acuity (CDVA) was20/20 OS, the refraction was +1.50 DC × 115 OS, and the HOA total was decreased to 0.077 by iTrace. The only factor that affected vision after operation was corneal astigmatism derived from suture, which could be corrected. The girl could acquire a clear optotype with glasses (Fig. 5). After the first week post-operation, her CDVA was 20/25, the refraction was +1.50 DC × 110 OD, the total HOA was 0.110, and the total LOA was 0.328. The CDVA was still 20/25 one month after operation, with total HOA at 0.110 and total LOA at 0.334. The refraction was +1.00 DC × 110—basically the same outcome as at the first week after operation. The position of IOL remained centered in the pupil region, as shown in Figure 6. This girl was satisfied with the clear visual quality of her sight.

## 3 Discussion

Microspherophakia is usually characterized by small and spherical lens.^[4,5,8]^ In normal people, the equatorial diameter of the lens grows from 6.5 mm at birth to about 9.0 mm by the age of 15 and remains at about 9.0 mm throughout life. The sagittal diameter develops to approximately 4.75 to 5.0 mm by the age of 80 to 90 years.^[9]^ The equatorial diameter and lens thickness of the OS we reported were 6.30 mm and 5.05 mm, respectively. The lens trembled with eye movement and the weak zonules all around could be clearly observed after pupillary dilation.

Microspherophakia is always combined with systemic disorders such as Weill-Marchesani syndrome (WMS),^[3,4,9–12]^ Marfan syndrome,^[3,4,11,12]^ hyperlysinaemia,^[10]^ Alport syndrome,^[3,8]^ Lowe's syndrome,^[8,13]^ Klinefelter syndrome,^[3]^ Peter's anomaly,^[8]^ mandibulofacial dysostosis,^[3,14]^ cri-du-chat syndrome,^[15]^ and chondrodysplasia punctate.^[16]^ Our patient had no clinical features associated with these syndromes. Her physical development, aside from her eyes and mental status, was normal.

The development of microspherophakia always combines with secondary glaucoma.^[7,17]^ Because of forward movement of the lens caused by weak and long zonules, pupillary block leads to acute angle closure.^[18]^ Continuous pupillary block can initiate synechial angle closure and trabecular meshwork destruction.^[17,18]^ Because of persistent forward movement of the lens-iris diaphragm, the IOP increase cannot be solved by laser iridotomy and medical therapy. Whereas reduction of IOP was confirmed after clear lens extraction.

Indications for clear lens extraction in patients with microspherophakia are corneolenticular contact, unilateral high myopia, pupillary block, and secondary intractable glaucoma.^[19]^ Lens surgery is not always the first choice, because of age, risk of zonular defects, small capsular bag for IOL, and postoperative visual problems.^[20]^ Being nagged by high myopia before secondary glaucoma, microspherophakia patients turn to wearing glasses or rigid gas permeable (RGP) contact lenses.

**Figure 7:**
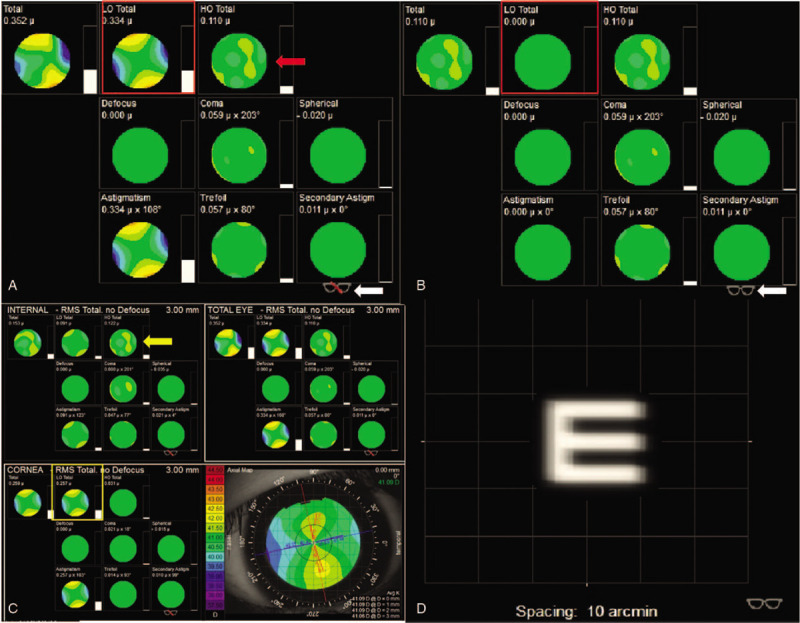
The iTrace report 1 month after operation. (A) Shows the status of correcting defocus (highmyopia), marked by the white arrow. Total low-order aberrations (LOA) was 0.334, indicated by the red square; and total high-order aberrations (HOA) was 0.110, indicated by the red arrow. (B) Shows the status of correcting total LOA, indicated by the white arrow. Total LOA was 0, indicated by the red square. (C) Shows the total LOA mainly derived from the cornea, indicated by the yellow square. The total HOA mainly derived from intraocular variation is indicated by the yellow arrow. (D) Shows the clear visual quality of correct LOA achieved by wearing glasses.

In our case, the anterior chamber depth was as deep as 3.40 mm OS and the peripheral anterior angle was open all around. There is no sign of glaucoma currently. However, the visual quality was still terrible, with HOA high up to 0.659 after collecting LOA (astigmatism and high myopia) by iTrace report. Wearing glasses or RGP could make few contributions to solve blurred vision (Fig. 3). The fundus examination and optical coherence tomography showed no obvious abnormity in Figure 2. The potential source of undesirable preoperative visual quality could be analyzed by iTrace. The mainly undesirable influence factor was from internal marked by yellow box in Figure 4. The patient would acquire a good visual quality by removing the internal factor with the residual factor from cornea after correcting the LOA. The total corneal HOA was as low as 0.056. On the contrary, the poor visual quality was mainly due to the internal high HOA (Figure 4), which is primarily contributed by the microspherophakia. Clear lens extraction via ultrasonic phacoemulsification was chosen as the therapy for this girl. The outcome was satisfactory for the patient, who achieved CDVA of 20/25, total HOA low of 0.110, and total LOA of 0.334 by iTrace. She could achieve clear vision through correcting astigmatism derived from suture; by wearing glasses, she obtained clearer vision 1 month after operation (Fig. 7). Clear lens extraction by ultrasonic phacoemulsification could offer the patient a better visual quality, as confirmed by the iTrace report.

**Figure 8:**
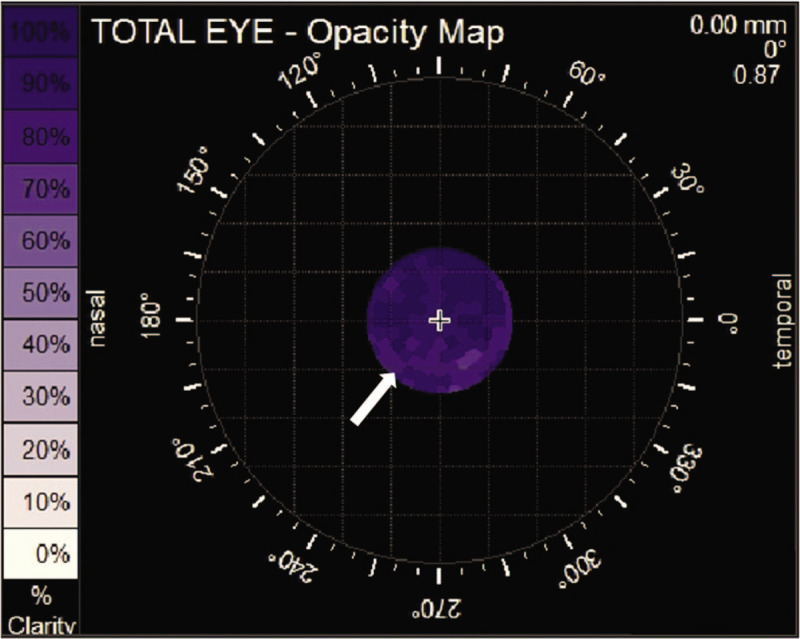
The iTrace report 1 month after operation. The white spots marked by the white arrow indicate posterior capsule regrowth and opacification.

We could analyse that the postoperative astigmatism might come from suture of the incision by iTrace. Because the postoperative LOA (marked with red box in Fig. 7A) mainly derived from cornea astigmatism (marked with yellow box in Fig. 7C), it is expected to lower after the stitches are removed and also could be solved by correcting LOA (marked white arrow in Fig. 7A and B). The total LOA was as low as 0 as shown in Figure B with red box and the visual quality was shown as “E” in Figure 7D. The mild rise of total HOA (marked with red arrow in Fig. 7A) 1 month after operation came from intraocular variation as showed in Figure 7C marked by yellow arrow, caused by posterior capsule regrowth and opacification as showed in Figure 8. This is a common complication which could be solved by membranectomy.^[20]^ The total HOA, total LOA, and the status of refraction were consistent between 1 week and 1 month post-operation, which indicated that the visual quality was stabilizing.

Meanwhile, clear lens extraction via ultrasonic phacoemulsification was a highly difficult surgery for microspherophakia. The operation process was complicated and high-risk which required extensive experience and great skill in cataract surgery. Farat et al reported a case in which they tried to implant IOL in a capsule bag, but they failed because the bag was too small to accommodate the IOL. The pressure of the lens haptics ovalized the capsular bag.^[21]^ Earlier reports advised lensectomy for microspherophakia, based on worries concerning the uncertainty of the IOL position derived from the weakness of the zonules.^[22]^ We choose technology to incarcerate the IOL optic region by anterior capsule opening, which played an important role in keeping the IOL steadily centered 1 month after operation. Femtosecond laser assisted surgery could be an more safe option for adult patient.^[23]^

Visual quality could be analyzed by iTrace via separating the source of influence factor into cornea, internal, and total. We can intuitively obtain the impact from each part, and then predict the postoperative outcome. ITrace could be a valuable auxiliary examination for the surgeon to make a decision of surgical intervention for microspherophakia patient.

## Acknowledgments

Authors are grateful to the patient and her parents, who gave their informed consent for publication.

## Author contributions

**Conceptualization:** Qian Liu, Xiaogang Wang, Suhua Zhang.

**Data curation:** Qian Liu.

**Formal analysis:** Qian Liu, Suhua Zhang.

**Investigation:** Qian Liu, Suhua Zhang.

**Methodology:** Qian Liu, Xiaogang Wang, Suhua Zhang.

**Project administration:** Qian Liu, Xiaogang Wang, Suhua Zhang.

**Resources:** Qian Liu, Suhua Zhang.

**Supervision:** Qian Liu, Xiaogang Wang, Suhua Zhang.

**Validation:** Qian Liu, Xiaogang Wang, Suhua Zhang

**Visualization:** Qian Liu, Xiaogang Wang.

**Writing – original draft:** Qian Liu.

**Writing – review & editing:** Qian Liu, Xiaogang Wang, Suhua Zhang.
